# Construction and validation of a nomogram to predict overall survival in patients with inflammatory breast cancer

**DOI:** 10.1002/cam4.2470

**Published:** 2019-08-12

**Authors:** Jian‐dong Diao, Li‐xia Ma, Mei‐yang Sun, Chun‐jiao Wu, Li‐juan Wang, Yan‐ling Liu, Yong‐jing Yang

**Affiliations:** ^1^ Department of Oncology and Hematology China‐Japan Union Hospital of Jilin University Changchun China; ^2^ Department of Oncology Jilin Cancer Hospital Changchun China; ^3^ Department of Breast Surgery Jilin Cancer Hospital Changchun China; ^4^ Department of Oncology The People's Hospital of Dehui City Changchun China; ^5^ Department of Radiation Oncology Jilin Cancer Hospital Changchun China

**Keywords:** inflammatory breast cancer, nomogram, overall survival, SEER database

## Abstract

In the present study, we examined the factors affecting survival of women with inflammatory breast cancer (IBC) and constructed and validated a nomogram to predict overall survival (OS) in these patients. The cohort was selected from the Surveillance, Epidemiology, and End Results (SEER) program between 1 January 2004 and 31 December 2013. Univariate and multivariate Cox proportional hazards regression models were constructed. A nomogram was developed based on significant prognostic indicators of OS. The discriminatory and predictive capacities of the nomogram were assessed using Harrell's concordance index (C‐index) and calibration plots. A total of 1651 eligible patients were identified, with a median survival time of 31 months (range 0‐131 months), and the 3‐ and 5‐year OS rates were 52.8% and 39.5%, respectively. Multivariate analysis revealed that race (*P* < .001), marital status (*P* = .011), N stage (*P* = .002), M stage (*P* < .001), hormone receptor (*P* < .001), human epidermal growth factor receptor‐2 (HER2) (*P* = .001), surgery (*P* < .001), chemotherapy (*P* < .001), and radiotherapy (*P* = .010) were independent prognostic indicators of IBC. These nine variables were incorporated to construct a nomogram. The C‐indexes of the nomogram were 0.738 (95% confidence interval [CI]: 0.717, 0.759) and 0.741 (95% CI: 0.717, 0.765) for the internal and external validations, respectively. The nomogram had a better discriminatory capacity for predicting OS than did the SEER summary stage (*P* < .001) or the American Joint Committee on Cancer tumor‐node metastasis staging systems (8th edition; *P* < .001). The calibration plot revealed satisfactory agreement between the findings and predicted outcomes in both the internal and external validations. The nomogram‐based 3‐ and 5‐year OS predictions for patients with IBC exhibited superior accuracy over the existing models.

## Graphical abstract

The present study aimed to examine the factors affecting survival of female patients with inflammatory breast cancer (IBC), as well as to construct and validate a nomogram to predict overall survival in IBC.

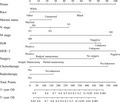

## 1 INTRODUCTION

Inflammatory breast cancer (IBC) is a rare and aggressive clinicopathological entity of breast cancer (BC), accounting for 1%‐6% of all BCs.^1^ IBC is classified as T4d in the tumor‐node metastasis (TNM) BC staging classification, which is clinically featured by a diffused duration on the skin with erysipeloid edges, generally without an underlying mass.^2^ Because IBC is rare, data on IBC are mainly acquired from small, single‐center, retrospective research studies or extrapolated from randomized prospective studies or the clinical experience of non‐IBC patients.^3^

TNM staging is a common tool for predicting the outcomes of cancer patients by evaluating the tumor size and location (T), local lymph node involvement (N), and distant metastasis (M).^4^ However, TNM classification alone is insufficient to encompass cancer biology or predict the outcomes of all BC cases, especially IBC.^5^ Furthermore, other clinicopathological factors, such as race, grade, adjuvant treatments, and molecular characteristics, can influence the prognoses of IBC patients.^6^, ^7^

The nomogram, a simple user‐friendly method of statistical prediction, compares favorably to the traditional TNM staging system in multiple cancers.^8^, ^9^, ^10^, ^11^, ^12^ However, no study has constructed a nomogram for IBC until now. In this study, we used data from the Surveillance, Epidemiology, and End Results (SEER) database to identify patient and tumor characteristics that affect the survival outcomes of women with IBC and subsequently construct a nomogram.

## 2 MATERIALS AND METHODS

### 2.1 Ethics statement

The National Cancer Institute's SEER program, initiated in 1973 and annually updated, uses population‐based data to develop comprehensive sources,^13^ covering approximately 30% of the US population in several geographic regions.^14^ For this study, we signed the SEER research data agreement to access SEER information, using reference number 16462‐Nov2016. Data were obtained following the approved guidelines. The Office for Human Research Protection considered this research to be on nonhuman subjects because the subjects were patients who had been researched by the United States Department of Health and Human Services and were publicly accessible and de‐identified. Thus, no institutional review board approval was required.

### 2.2 Study population

Patient data were acquired from the SEER database (Submission, November 2016). The SEER*State v8.3.5 tool, released on 6 March 2018, was employed to select and identify eligible patients. The study duration ranged from 1 January 2004 to 31 December 2013. The inclusion criteria for data screening were as follows: (a) age at diagnosis ≥ 20 years; (b) women with primary IBC; and (c) IBC diagnosis was consistent with the International Classification of Disease for Oncology, third edition (coded as 8530/3).The exclusion criteria were as follows: (a) patients under 20 years old; (b) patients had more than one primary malignancy; (c) incomplete or unavailable survival data; (d) patients had only a clinical diagnosis; (e) inaccessible critical clinicopathological data, including marital status, race, 8th American Joint Committee on Cancer (AJCC) tumor stage, and surgical information;(f) patient died within 3 months after surgery; and (g) patients without prognostic data. Eligible patients were enrolled as the SEER primary cohort. Patients from five randomly selected registries (Alaska Natives, Atlanta, California, Detroit, and Greater Georgia) were assigned to the validation cohort, while the remaining patients were assigned to the training cohort.

### 2.3 Covariates and endpoint

The following 12 clinicopathological variables were analyzed: age (<40, 40‐49, 50‐59, 60‐75, or > 75 years), race (white, black, or other), marital status (married or unmarried), grade (grade I/II, grade III/IV, or unknown), N stage (N0, N1, N2, or N3), M stage (M0 or M1), tumor extension (<50%, >50%, or unknown), hormone receptor (HoR; negative, positive, or unknown), HER‐2 (negative, positive, or unknown), surgery (no surgery, partial mastectomy, simple mastectomy, or radical mastectomy), chemotherapy (no/unknown or yes), and radiotherapy(no/unknown or yes).Widowed, separated, divorced, or single (having a domestic partner or never married) patients were classified as unmarried. Age was further converted into categorical variables according to the recognized cutoff values. All eligible cases were regrouped according to the 8th AJCC TNM staging system. Tumor extension was defined as the percentage of tumor area in the affected unilateral breast. The classification of tumor HoR was as follows: HoR‐positive (at least one positive outcome for estrogen receptor [ER] or progesterone receptor [PR]) or HoR‐negative (both negative outcomes for ER and PR). ER/PR‐positive disease was defined as positive staining in 1% or more of the cells.^15^

The primary endpoint in this study was overall survival (OS). OS was defined as the duration from diagnosis to the most recent follow‐up date or date of death. The predetermined cutoff date was 31 December 2014, because the SEER 2016 submission database contains death information until 2014.

### 2.4 Statistical analysis

#### 2.4.1 Nomogram construction

Categorical variables were presented as frequencies and proportions and were compared using a chi‐squared test. Univariate prognostic analysis was conducted using the Kaplan‐Meier method and log‐rank test. Significant prognostic factors identified from the univariate analysis were further analyzed in a multivariate Cox proportional hazards model along with the corresponding 95% confidence interval (CI) for each potential risk factor. Afterward, a nomogram model was constructed based on the training cohort to predict 3‐ and 5‐year OS by including all independent prognostic factors using the rms package in R software version 3.51.

#### 2.4.2 Nomogram validation

The nomogram was validated based mainly on the internal (training cohort) and external (validation cohort) discrimination and calibration measurements. The concordance index (C‐index) was used to evaluate the discriminative capacity of the nomogram, which mainly measured the differences between predicted and actual outcomes.^16^ A higher C‐index suggested a superior discriminative capacity for survival outcomes. The Rcorrp.cens package in Hmisc in R was used to compare the nomogram with the SEER summary stage or TNM 8th staging classification, followed by C‐index evaluation. Calibration plots were used to construct marginal estimates vs the model, indicating the calibration between nomogram‐predicted and actual survival. A calibration plot along the 45‐degree line implicated a perfect model, with great consistency between the predicted and actual outcomes. SPSS software, version 19.0 (SPSS Inc, Chicago, IL, USA) and R software, version 3.51 (http://www.r-project.org) were used for statistical analysis, and *P* < .05 was considered statistically significant.

## 3 RESULTS

### 3.1 Patient screening process

**Figure 1:**
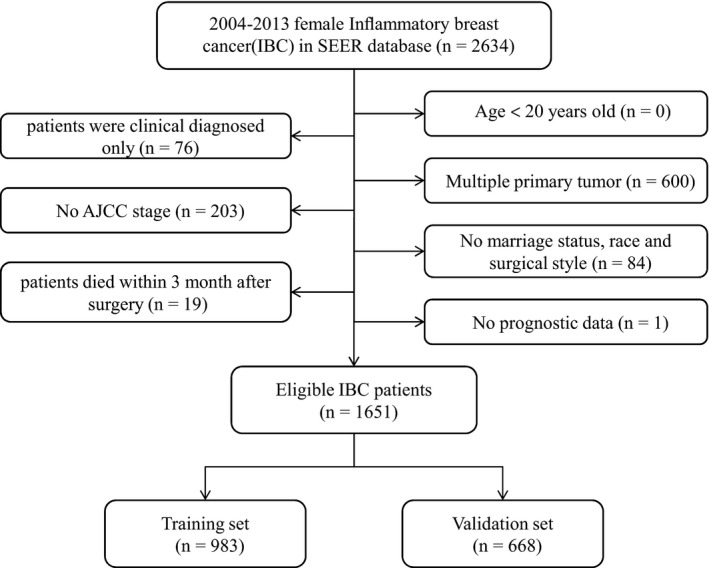
Flow chart for screening eligible patients. Abbreviations: AJCC, American Joint Committee on Cancer; SEER, Surveillance, Epidemiology, and End Results

In total, 1651 eligible women diagnosed with IBC from January 2004 to December 2013 were included in the study. Figure 1 shows the specific screening process. The median follow‐up was 31 months, ranging from 0 to 131 months. The median age at diagnosis was 56 years (range, 22‐98 years). Among all patients, 983 and 668 subjects were assigned to the training and validation cohorts, respectively. Table 1 lists the demographic and clinicopathological features, and no statistically significant differences were found between the two groups.

**Table 1:** Patient demographics and pathological characteristics

Variables	All patients (n = 1651)	Training set (n = 983)	Validation set (n = 668)	*P*‐value^b^
	N (%)	N (%)	N (%)	
Age (y)				.322
<40	177 (10.72)	101 (10.27)	76 (11.38)	
40‐49	322 (19.50)	187 (19.02)	135 (20.21)	
50‐59	481 (29.13)	289 (29.40)	192 (28.74)	
60‐75	484 (29.32)	282 (28.69)	202 (30.24)	
≥75	187 (11.33)	124 (12.61)	63 (9.43)	
Race				.227
White	1290 (78.13)	782 (79.55)	508 (76.05)	
Black	270 (16.35)	149 (15.16)	121 (18.11)	
Other^a^	91 (5.51)	52 (5.29)	39 (5.84)	
Marital status				.833
Married	848 (51.36)	507 (51.58)	341 (51.05)	
Unmarried	803 (48.64)	476 (48.42)	327 (48.95)	
Grade				.196
Grade I/II	345 (20.90)	220 (22.38)	125 (18.71)	
Grade III/IV	928 (56.21)	541 (55.04)	387 (57.93)	
Unknown	378 (22.90)	222 (22.58)	156 (23.35)	
N stage				.773
N0	287 (17.38)	167 (16.99)	120 (17.96)	
N1	684 (41.43)	414 (42.12)	270 (40.42)	
N2	294 (17.81)	169 (17.19)	125 (18.71)	
N3	386 (23.38)	233 (23.70)	153 (22.90)	
M stage				.300
M0	1153 (69.84)	677 (68.87)	476 (71.26)	
M1	498 (30.16)	306 (31.13)	192 (28.74)	
tumor extension				.126
<50	850 (51.48)	506 (51.48)	344 (51.50)	
≥50	378 (22.90)	211 (21.46)	167 (25.00)	
Unknown	423 (25.62)	266 (27.06)	157 (23.50)	
HoR				.068
Negative	726 (43.97)	426 (43.34)	300 (44.91)	
Positive	798 (48.33)	492 (50.05)	306 (45.81)	
Unknown	127 (7.69)	65 (6.61)	62 (9.28)	
HER‐2				.240
Negative	280 (16.96)	175 (17.80)	105 (15.72)	
Positive	167 (10.12)	106 (10.78)	61 (9.13)	
Unknown	1204 (72.93)	702 (71.41)	502 (75.15)	
Surgery				.708
No surgery	476 (28.83)	275 (27.98)	201 (30.09)	
Partial mastectomy	72 (4.36)	45 (4.58)	27 (4.04)	
Simple mastectomy	177 (10.72)	110 (11.19)	67 (10.03)	
Radical mastectomy	926 (56.09)	553 (56.26)	373 (55.84)	
Chemotherapy				.824
No/unknown	231 (13.99)	136 (13.84)	95 (14.22)	
Yes	1420 (86.01)	847 (86.16)	573 (85.78)	
Radiotherapy				.236
No/unknown	830 (50.27)	506 (51.48)	324 (48.50)	
Yes	821 (49.73)	477 (48.52)	344 (51.50)	

Abbreviation: HoR, hormone receptor.

a Other includes American Indian/Alaska native, Asian/Pacific Islander, and unknown.

b The comparison results between training set and validation set.

### 3.2 Nomogram construction

**Figure 2:**
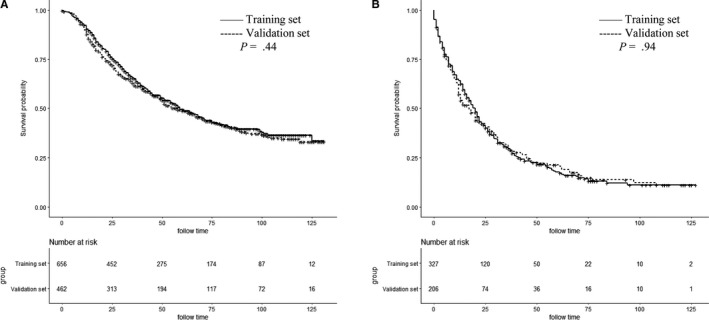
Kaplan‐Meier survival plots of the patients with inflammatory breast cancer in the training set and in the validation set for regional (A) and distant disease (B)

**Figure 3:**
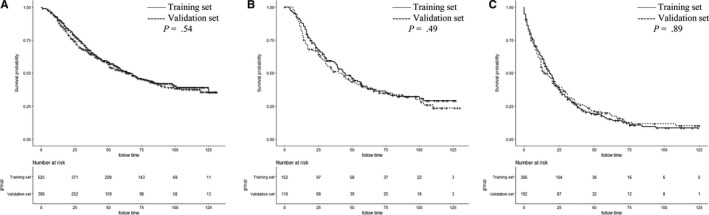
Kaplan‐Meier survival plots of the patients with inflammatory breast cancer in the training set and in the validation set for American Joint Committee on Cancer stage IIIA (A), IIIB (B), and IV (C)

**Figure 4:**
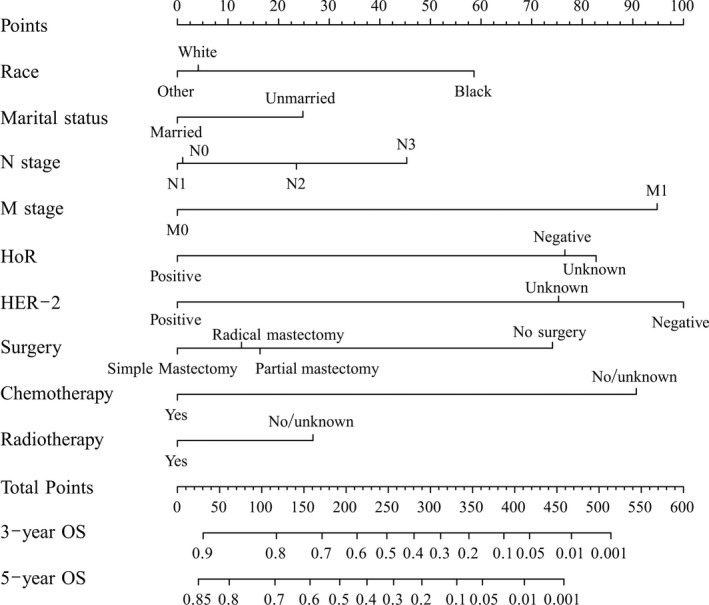
A nomogram for predicting 3‐ and 5‐year overall survival (OS) of patients with inflammatory breast cancer. Abbreviations: HoR, hormone receptor

The 3‐ and 5‐year OS rates were 52.8% and 39.5%, respectively. Figure 2 shows the OS curves for localized, regional, and distant diseases. Figure 3 shows the OS survival curves for AJCC stages IIIA, IIIB, and IV disease. Table 2 lists the independent factors that significantly influenced OS in the multivariate analysis. Nine factors remained as independent factors after adjusting for other risk factors, including race (*P* < .001), marital status (*P* = .011), N stage (*P* = .002), M stage (*P* < .001), HoR (*P* < .001), human epidermal growth factor receptor‐2 (HER2) (*P* = .001), surgery (*P* < .001), chemotherapy (*P* < .001), and radiotherapy (*P* = .010). These nine independent factors in the training cohort were incorporated into a nomogram‐based prediction of 3‐ and 5‐year OS rates (Figure 4). The nomogram showed that HER2 status and M stage contributed the most to the prognosis, followed by chemotherapy, HoR, surgery, N stage, radiotherapy, and marital status. The survival probability of each patient could be easily calculated by adding the scores for every variable.

**Table 2:** Univariate and multivariate analyses of overall survival in the training set

Variables	Univariate analysis	Multivariate analysis	
	*P*‐value	HR (95% CI)	*P*‐value
Age (years)	<.001		.288
˂40		Reference	
40‐49		1.111 (0.795, 1.554)	.537
50‐59		1.082 (0.793, 1.476)	.620
60‐75		1.088 (0.796, 1.489)	.596
>75		1.423 (0.991, 2.042)	.056
Race	<.001		<.001
White		Reference	
Black		1.586 (1.285, 1.957)	<.001
Other^a^		0.962 (0.655, 1.413)	.843
Marital status	<.001		.011
Married		Reference	
Unmarried		1.235 (1.049, 1.454)	
Grade	.021		.258
Grade I/II		Reference	
Grade III/IV		1.091 (0.881, 1.350)	
Unknown		0.914 (0.709, 1.179)	
N stage	.047		.002
N0		Reference	
N1		0.993 (0.779, 1.265)	.954
N2		1.213 (0.909, 1.618)	.190
N3		1.460 (1.122, 1.901)	.005
M stage	<.001		<.001
M0		Reference	
M1		2.248 (1.850, 2.733)	
Tumor extension^b^	.087		.140
<50%		Reference	
≥50%		1.215 (0.999, 1.479)	.052
Unknown		1.016 (0.731, 1.412)	.923
HoR	<.001		<.001
Negative		Reference	
Positive		0.521 (0.437, 0.621)	<.001
Unknown		1.048 (0.764, 1.438)	.771
HER, 2	.001		.001
Negative		Reference	
Positive		0.425 (0.274, 0.661)	<.001
Unknown		0.810 (0.636, 1.030)	.085
Surgery	<.001		<.001
No surgery		Reference	
Partial mastectomy		0.613 (0.407, 0.922)	.019
Simple mastectomy		0.532 (0.386, 0.733)	<.001
Radical mastectomy		0.592 (0.472, 0.743)	<.001
Chemotherapy	<.001		<.001
No/unknown		Reference	
Yes		0.464 (0.370, 0.583)	
Radiotherapy	<.001		.010
No/unknown		Reference	
Yes		0.794 (0.666, 0.946)	

Abbreviation: HoR, hormone receptor.

a Other includes American Indian/Alaska native, Asian/Pacific Islander.

b Tumor extension means the percentage of the area of tumors in the unilateral breast.

### 3.3 Nomogram validation

**Figure 5:**
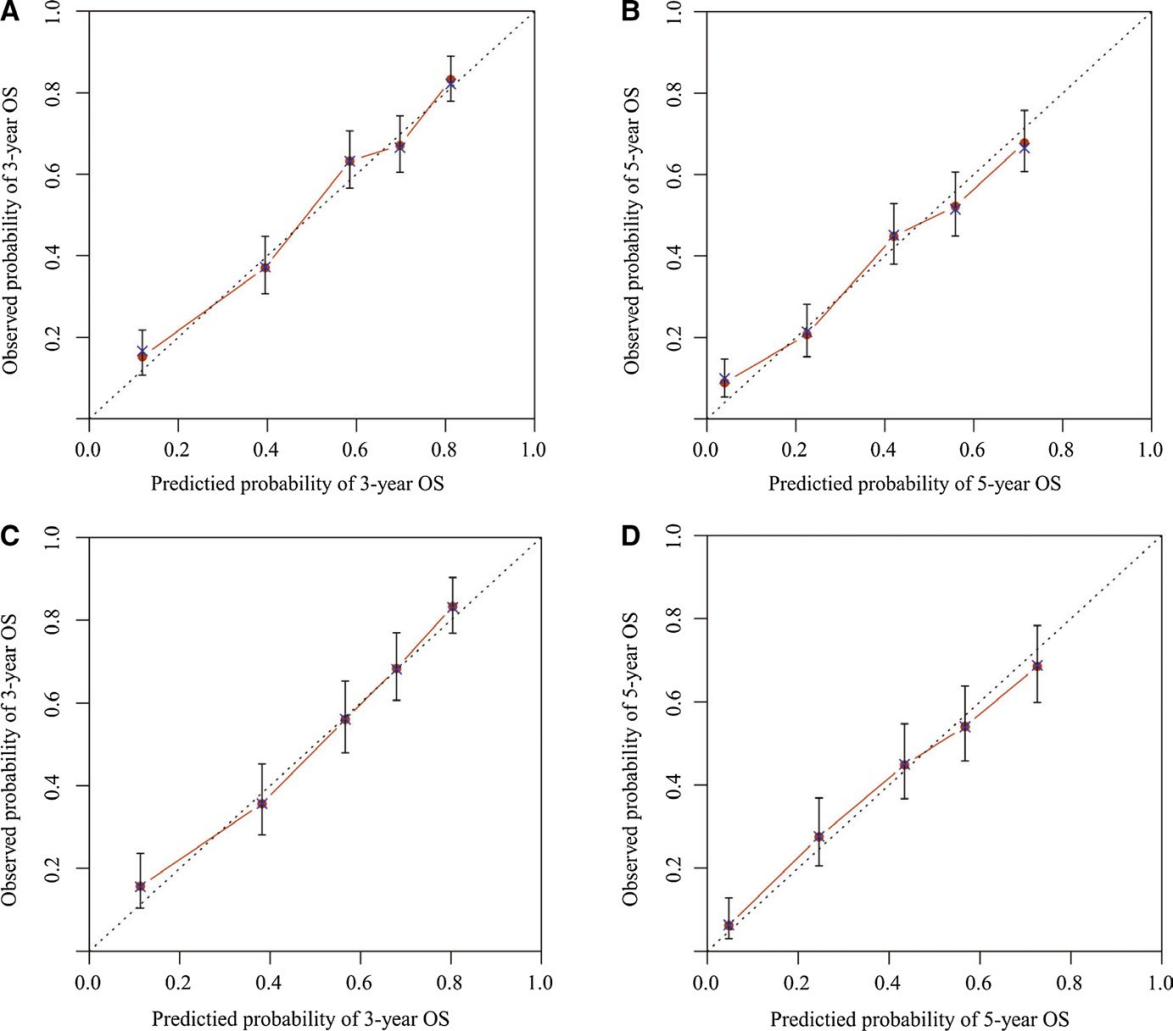
Calibration plots of the nomogram for 3‐ and 5‐year overall survival (OS) (A,B) prediction in the training set, and 3‐ and 5‐year OS (C,D) prediction in the validation set. The *X*‐axis represents the nomogram‐predicted probability of survival; the *Y*‐axis represents the actual OS probability. Plots along the 45‐degree line indicate a perfect calibration model in which the predicted probabilities are identical to the actual outcomes. Vertical bars indicate 95% confidence intervals

The nomograms were internally and externally validated. The C‐indexes for OS prediction in the nomogram were 0.738 (95% CI: 0.717, 0.759) and 0.741 (95% CI: 0.717, 0.765) for the training (internal validation) and validation (external validation) cohorts, respectively. Moreover, the discriminative capacity of the nomogram was compared with that of the SEER summary stage and TNM 8th staging classification, which revealed that the nomogram was significantly superior to the SEER and TNM 8th edition staging classification in both the training and validation sets (*P* < .001; Table 3). Finally, the internal and external calibration plots of the nomogram showed good agreement between the nomogram‐based predictions and actual outcomes (Figure 5).

**Table 3:** C‐indexes for the nomogram and other stage systems in patients with inflammatory breast cancer

Classification	Training set		Validation set	
	C‐index (95% CI)	*P*‐value^a^	C‐index (95% CI)	*P*‐value^a^
Nomogram	0.738 (0.717, 0.759)		0.741 (0.717, 0.765)	
AJCC eighth stage	0.648 (0.627, 0.669)	<.001	0.636 (0.611, 0.661)	<.001
SEER summary stage	0.630 (0.610, 0.650)	<.001	0.617 (0.593, 0.641)	<.001

Abbreviations: AJCC, American Joint Committee on Cancer; C‐index, concordance index; CI, confidence interval; HR, hazard ratio; SEER, Surveillance, Epidemiology, and End Results.

a All are compared with nomogram.

## 4 DISCUSSION

In total, 1651 IBC patients from the SEER database were analyzed. The constructed nomogram successfully predicted the 3‐ and 5‐year OS of IBC patients, demonstrating favorable discrimination and calibrations, which were internally and externally validated. Additionally, the nomogram demonstrated better prediction capacity than that of the SEER summary stage or TNM 8th edition staging classification.

Currently, IBC has no established risk factors. However, many epidemiological studies have clarified the characteristics of IBC.^3^ Of these, the most important suspected risk factors associated with IBC include race, body mass index, and age.^7^ Wu et al found that BC subtype is clinically useful for predicting survival in IBC. Patients with the HoR‐/HER2‐subtype had significantly poorer OS than did the other three subtypes.^17^ Positive node involvement is also an adverse prognostic factor. In addition, ER/PR positivity and therapeutic approaches, including surgical resection and radiotherapy in node‐positive patients, have been reported to enhance outcomes.^18^ In our study, we found nine independent prognostic factors of OS, including race, marital status, N stage, M stage, HoR, HER2, surgery, chemotherapy, and radiotherapy.

IBC has historically been treated with surgery and/or radiotherapy; however, the 5‐year OS is under 5%.^18^ Before 1950, the median survival for patients treated by mastectomy was 19 months, and none of these patients survived to 5 years.^6^ Administering definitive radiotherapy without surgery showed a 5‐year survival rate without recurrence of 17% and an OS rate of 28%. Combining surgery and radiotherapy improves OS.^19^ Moreover, the introduction of systemic chemotherapy showed an additional survival benefit.^20^ Thus, trimodal therapy, including chemotherapy, surgery, and radiotherapy, has gradually become the standard of care for IBC. This therapy was established at the First International Conference on IBC in December 2008 to manage IBC.^21^ Our study also found that surgery, chemotherapy, and radiotherapy significantly prolonged patient survival, confirming the effectiveness of trimodal therapy.

Nomograms are a user‐friendly statistical method that can estimate survival or a specific outcome through simple graphical presentation.^22^ Moreover, nomograms can better predict outcomes than conventional AJCC TNM staging can for some malignant tumors and are recognized as an alternative or novel standard.^23^, ^24^ Additionally, nomograms can facilitate decision‐making under complicated clinical conditions without needing standard guidelines.^25^, ^26^

This study has several strengths. The clinicopathological data on IBC patients collected from the SEER dataset were detailed, thus helping to ensure the accuracy of our constructed nomogram. Moreover, our nomogram demonstrates superior discriminative power for predicting OS over the SEER or TNM eighth edition staging classification. Calibration was used to confirm the validity and presentation of the nomogram. Easily accessible clinicopathological factors were used, which were convenient for clinical application of the nomogram.

Our study had some limitations. First, the nomogram was established retrospectively using the SEER database, which may lead to potential selection bias. Second, some prognosis‐related clinicopathological factors were inaccessible in the SEER database, including vascular invasion and the specific radiotherapy and chemotherapy contents, which will be a main focus in future studies. Finally, as a user‐friendly method for decision‐making, some prognostic variables were not included in the nomogram; thus, the nomogram may not always yield accurate prognoses in clinical practice.

## 5 CONCLUSION

In summary, our study was the first to construct a well‐validated nomogram for women with IBC. This nomogram may help clinicians identify patients at a high risk of overall mortality within 3‐5 years. However, the unknown prognostic factors must be further exploited to optimize the nomogram, and more external validation is required.

## Data Availability

The data that support the findings of this study are available from the corresponding author upon reasonable request.
